# SARS-CoV-2 infection is associated with anti-desmoglein 2 autoantibody detection

**DOI:** 10.1093/cei/uxad046

**Published:** 2023-04-24

**Authors:** Kerensa E Ward, Lora Steadman, Abid R Karim, Gary M Reynolds, Matthew Pugh, Winnie Chua, Sian E Faustini, Tonny Veenith, Ryan S Thwaites, Peter J M Openshaw, Mark T Drayson, Adrian M Shields, Adam F Cunningham, David C Wraith, Alex G Richter

**Affiliations:** Institute of Immunology and Immunotherapy, University of Birmingham, Birmingham, West Midlands, UK; Institute of Immunology and Immunotherapy, University of Birmingham, Birmingham, West Midlands, UK; Institute of Immunology and Immunotherapy, University of Birmingham, Birmingham, West Midlands, UK; Centre for Liver and Gastrointestinal Research, Institute of Immunology and Immunotherapy, University of Birmingham, Birmingham, West Midlands, UK; Institute of Cancer and Genomic Sciences, University of Birmingham, Birmingham, West Midlands, UK; Institute of Cardiovascular Sciences, University of Birmingham, Birmingham, West Midlands, UK; Institute of Immunology and Immunotherapy, University of Birmingham, Birmingham, West Midlands, UK; Department of Critical Care, University Hospitals Birmingham NHS Foundation Trust, Birmingham, UK; National Heart and Lung Institute, Imperial College London, London, UK; National Heart and Lung Institute, Imperial College London, London, UK; Institute of Immunology and Immunotherapy, University of Birmingham, Birmingham, West Midlands, UK; Institute of Immunology and Immunotherapy, University of Birmingham, Birmingham, West Midlands, UK; Department of Clinical Immunology, University Hospitals Birmingham NHS Foundation Trust, Birmingham, UK; Institute of Microbiology and Infection, University of Birmingham, Birmingham, UK; Institute of Immunology and Immunotherapy, University of Birmingham, Birmingham, West Midlands, UK; Institute of Immunology and Immunotherapy, University of Birmingham, Birmingham, West Midlands, UK; Department of Clinical Immunology, University Hospitals Birmingham NHS Foundation Trust, Birmingham, UK

**Keywords:** autoantibodies, autoimmunity, infection, virus

## Abstract

Post-acute cardiac sequelae, following SARS-CoV-2 infection, are well recognized as complications of COVID-19. We have previously shown the persistence of autoantibodies against antigens in skin, muscle, and heart in individuals following severe COVID-19; the most common staining on skin tissue displayed an inter-cellular cement pattern consistent with antibodies against desmosomal proteins. Desmosomes play a critical role in maintaining the structural integrity of tissues. For this reason, we analyzed desmosomal protein levels and the presence of anti-desmoglein (DSG) 1, 2, and 3 antibodies in acute and convalescent sera from patients with COVID-19 of differing clinical severity. We find increased levels of DSG2 protein in sera from acute COVID-19 patients. Furthermore, we find that DSG2 autoantibody levels are increased significantly in convalescent sera following severe COVID-19 but not in hospitalized patients recovering from influenza infection or healthy controls. Levels of autoantibody in sera from patients with severe COVID-19 were comparable to levels in patients with non-COVID-19-associated cardiac disease, potentially identifying DSG2 autoantibodies as a novel biomarker for cardiac damage. To determine if there was any association between severe COVID-19 and DSG2, we stained post-mortem cardiac tissue from patients who died from COVID-19 infection. This confirmed DSG2 protein within the intercalated discs and disruption of the intercalated disc between cardiomyocytes in patients who died from COVID-19. Our results reveal the potential for DSG2 protein and autoimmunity to DSG2 to contribute to unexpected pathologies associated with COVID-19 infection.

## Teaser

We find raised levels of anti-DSG2 autoantibodies in sera from individuals following severe COVID-19. Staining of post-mortem cardiac tissue from individuals that died from COVID-19 with an anti-DSG2 antibody revealed disruption of the intercalated disc between cardiomyocytes that was consistent with separation of the DSG2 protein homodimer. Our results reveal the potential for DSG2 protein and autoimmunity to DSG2 to contribute to unexpected pathologies associated with COVID-19 infection.

## Introduction

Infection with SARS-CoV-2 has been associated with an array of unanticipated symptoms, both during the acute illness and in the immediate and long-term convalescence period. We have previously reported an increased rate of autoantibody detection in individuals post-SARS-CoV-2 infection [1]. Our previous observation that autoantibodies directed against autoantigens in the skin, skeletal muscle, and heart are overrepresented in individuals following severe COVID-19, in comparison to individuals admitted to intensive care for other reasons, suggests a disease-specific effect. Most commonly, skin autoantibodies exhibited an intercellular cement pattern by immunofluorescence microscopy, similar to findings in bullous pemphigus, a blistering autoimmune skin disease associated with autoantibodies against desmoglein (DSG) adhesion molecules [2].

The DSG family of proteins play an important role in maintaining tissue integrity through cell-to-cell adhesion within specialized protein complexes called desmosomes. The cadherin family of cellular adhesion molecules includes DSG and desmocollin, which through the junctional proteins plakoglobin and plakophilin help secure desmoplakin and keratin filaments to the desmosome structure [3]. Desmosomes are found in tissues that experience significant mechanical stress, such as cardiac muscle tissue, gastrointestinal mucosa, and skin epithelia and have a key role in maintaining cardiomyocyte and epithelial integrity as well as intestinal barrier function.

DSG1 and 3 are the predominant autoantibody targets found in pemphigus vulgaris (PV), a life-threatening autoimmune disease of the skin and mucous membranes. The study of PV provided the first link between desmosomes, DSGs, and disease, but further analyses are now beginning to identify a wider role for DSGs in human health [4].

DSG2 is found in all desmosome-bearing epithelial cells, skin, and cardiomyocytes. Whilst DSG2 is not necessary for cardiac development, it is required for mechanical integrity [5]. Desmosomes connect cardiomyocytes at their intercalated discs. An example of the importance of this linkage is demonstrated in arrhythmogenic right ventricular cardiomyopathy (ARVC) [6]. With intercalated disc disruption, there is an increase in fat deposition and scar tissue, and ultimately, this perturbation of gap junctions leads to an impaired capacity of cardiac action potentials to spread through cardiac tissue resulting in cardiac conduction delay and ventricular arrhythmias. Mutation in the DSG2 gene or autoantibodies against the protein [7] are both associated with ARVC [8, 9].

Cardiac complications are being increasingly described either during or as post-acute sequelae following SARS-CoV-2 infection [10, 11]. These include raised troponin in patients with severe disease [12], viral cardiomyopathy [12], and cardiac pathology on post-mortem hearts [13]. Given our previous findings of cardiac muscle autoantibodies [1] we hypothesized that SARS-CoV-2 infection may contribute to cardiac pathology through a direct or indirect autoimmune process.

In this study, we have measured desmosomal protein levels and investigated the presence of anti-DSG1, DSG2, and DSG3 antibodies in sera from patients with acute COVID-19 and in those who have recovered from COVID-19 infection. To determine if these autoantibodies are disease-specific, we have recruited three comparison cohorts, one with severe influenza infection, a healthy control cohort, and a cohort of patients with common underlying cardiac complications. Finally, we examine cardiac tissue, obtained post-mortem from patients who died from COVID-19, to explore the potential clinical relevance of the DSG2 autoantibodies and cardiac damage.

## Methods and materials

### Ethical approval

All investigations undertaken conformed to the principles outlined in the Declaration of Helsinki. Northwest-Preston Research Committee (ref: 20/NW/0240 IRAS Project ID: 282164) gave approval for the recruitment of 39 individuals in intensive therapy unit (ITU) with severe COVID-19 and 25 individuals in convalescence from admission to the ITU with severe COVID-19 infection. London-Camden and Kings Cross Research Ethics Committee (ref. 20/HRA/1817) gave approval for recruitment of individuals as part of the COvid-19 COnvalescent immunity (COCO) study. Outer West London Research Ethics Committee (ref: 09/MRE00/67) and the NHS National Research Ethics Service (ref: 09/H0709/52) gave approval for the influenza samples. Birmingham and Black Country atrial fibrillation registry (BBC-AF, IRAS ID 97753, REC reference 12/WM/0344) gave approval for the samples comprising the cardiac cohort. The NHS National Research Ethics Committee (ref: 19/NE/0336, IRAS ID 193937) gave approval for the post-mortem samples as part of the Long and Short-Term effects of Standard-of-Care treatments for Cancer (LoST-SoCC) study.

### Patient cohorts

**Figure 1.**
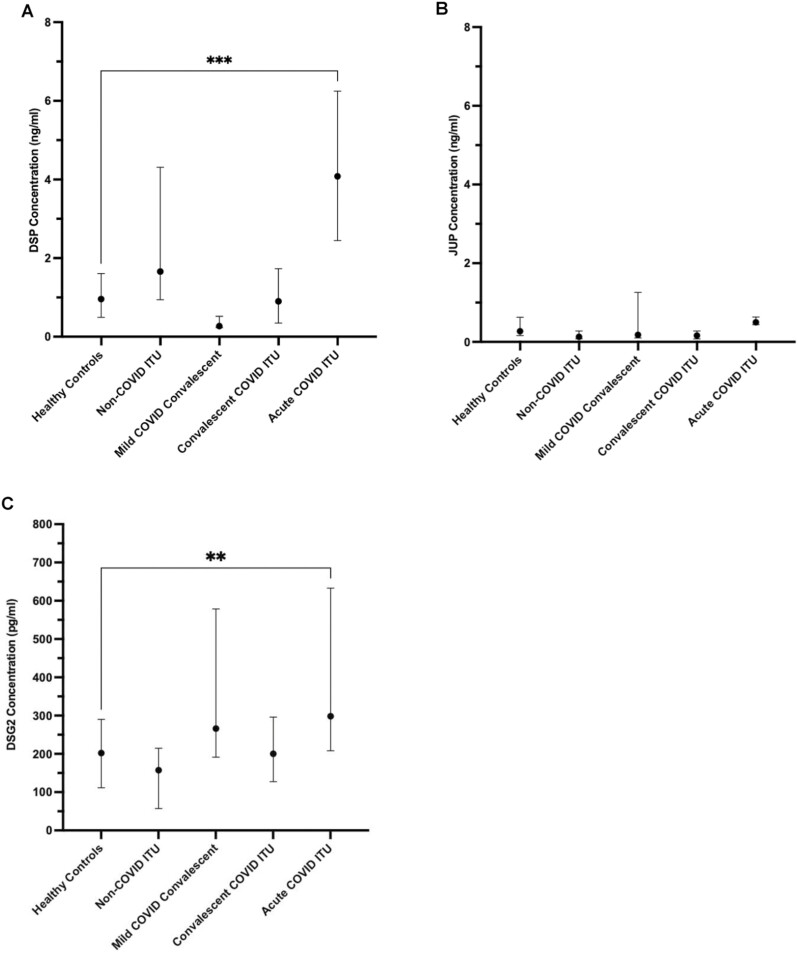
Desmosome proteins detected in serum following SARS-CoV-2 infection. Detection of (**A**) desmoplakin (DSP, ***P = 0.0004), (**B**) junction plakoglobin (JUP), and (**C**) desmoglein 2 (DSG2, **P = 0.0051) protein by ELISA. Results are expressed as median concentration with interquartile range, and all groups are statistically not significant from healthy controls unless annotated.

We recruited a cohort of 39 patients with severe COVID-19 on the ITU at the time of sample collection, and 25 patients that were in convalescence 3–6 months postdischarge from the ITU for COVID-19 infection (Ethics: Northwest-Preston Research Committee, ref. 20/NW/0240 IRAS Project ID: 282164). To determine whether there were different rates or patterns of infection following severe or non-hospitalized disease, we included a cohort of 40 health care workers from the COCO study approved by the London–Camden and Kings Cross Research Ethics Committee (ref. 20/HRA/1817) that had suffered from COVID-19 in the first wave in spring 2020 and had tested positive by PCR but had not been hospitalized. As controls for these COVID-19 cohorts, we recruited 16 individuals admitted to the ITU for any reason other than COVID-19. We also recruited two healthy control cohorts; first, sera provided by 44 healthy control subjects prior to the pandemic or by health care workers recruited from the COCO study. The samples from the COCO study were from subjects that were deemed to have never had a COVID-19 infection; they had suffered no clinical symptoms of COVID-19 in the first wave and were negative for IgG-, IgA-, and IgM- specific antibodies against the nucleocapsid and spike protein from SARS-CoV-2—these samples were used to examine levels of circulating desmosomal proteins in sera. Due to insufficient serum volumes being available in the above samples to perform both circulating desmosomal protein and autoantibody assessments, we also used sera from a second cohort of 50 COVID-19 naive COCO healthy controls for the DSG2 autoantibody ELISA testing (results shown in Fig. 1 and Table 1).

**Table 1.** Description of clinical cohorts and frequency of positivity for autoantibodies

	Healthy controls	Influenza	Non-COVID ITU	Mild COVID convalescent	Acute COVID ITU	Convalescent COVID ITU	Cardiac cohort
Total individuals, *n*	50	48	16	40	39	25	34
Age (years), median (IQR)	44 (37‐49)	42.5 (29‐51)	57 (46.5‐68.5)	44 (35‐51)	54 (50‐61)	55 (44.5‐61)	79 (71‐83)
Female,* n* (%)	34(68)	20 (42)	9 (56)	34 (85)	9 (23)	5 (20)	12 (35)
Pre-existing cardiac comorbidity, *n* (%)	0 (0)	6 (13)	4 (25)	4 (10)	22 (56)	9 (36)	34 (100)
Frequency of positivity for autoantibodies							
Skeletal muscle, *n* (%)	n/a	1 (2)	0 (0)	1 (3)	9 (23)	14 (30)	n/a
Cardiac, *n* (%)	n/a	3 (6)	1 (6)	0 (0)	2 (8)	10 (40)	0 (0)
Epidermal, *n* (%)	n/a	9 (19)	0 (0)	12 (30)	18 (46)	13 (52)	n/a

To compare results in COVID-19 patients with another group of individuals with severe respiratory viral illness requiring hospitalisation, we obtained acute sera from 42 individuals who had taken part in the Mechanisms of Severe Acute Influenza Consortium (MOSAIC) study which sampled patients hospitalized with influenza between 2009 and 2011 prior to the COVID-19 pandemic (NHS National Research Ethics Service, Outer West London REC (09/H0709/52, 09/MRE00/67)). To explore whether cardiac comorbidity, aside from ARVC, were associated with DSG2 antibody production, we obtained pre-pandemic sera from 34 patients from the Birmingham and Black Country Atrial Fibrillation registry (BBC-AF, IRAS ID 97753, REC reference 12/WM/0344) who had multiple cardiovascular comorbidities; heart failure (*n* = 28, 82%), atrial fibrillation (*n* = 25, 74%), hypertension (*n* = 12, 35%), coronary artery disease (*n* = 14, 41%), or myocardial infarction (*n* = 9, 26%). Characteristics of each of these cohorts, including previous history of cardiac disease, are described in Table 1. Post-mortem tissue, from cases of fatal COVID-19 and pre-pandemic controls without acute cardiac pathology, was collected as part of the LoST-SoCC study (IRAS 193937, Rec: 19/NE/0336). Post-mortems were conducted at the Royal Glamorgan Hospital and Morriston Hospital, South Wales following consent from families. All COVID-19 patients received a positive SARS-CoV-2 swab either in life or at post-mortem, and COVID-19 was given as the cause of death in all cases.

### Indirect immunofluorescence

Slides with sections of Monkey Heart (REF: S0206), Skeletal Muscle (REF: S0215) and Oesophagus (REF: S0205E) (Bio-Diagnostics Ltd, UK) were used to detect anti-cardiac, anti-skeletal muscle and epithelial autoantibodies, respectively. In short, for anti-cardiac antibodies patient sera, positive and negative controls were each diluted to 1/5 in PBS and 50 µl added onto sections. For anti-skeletal muscle and anti-epithelial antibodies, the samples were diluted 1/10 with PBS. Slides were incubated at room temperature for 30 minutes before rinsing with PBS and washing for 5 minutes. 30 µl of anti-human-FITC-IgG (monkey absorbed) conjugate (J502-5, BioDiagnostics Ltd, UK) was added to each well and incubated at room temperature for 30 minutes. A final PBS rinse and wash was conducted before mounting the slides onto coverslips and reading with a fluorescence microscope with a 495 nm exciter filter and 515 nm barrier filter.

### ELISAs

#### Commercial ELISAs used to detect serum desmosomal protein concentration

Human DSG1 (Invitrogen, Thermo Fisher Scientific, UK; CAT#EH153RB), human DSG2 (Invitrogen, CAT#EH154RB), human DSG3 (Invitrogen; CAT#EH155RB), human junctional plakoglobin (JUP) (Biorbyt; REF: orb563210) and human desmoplakin (DSP) ELISA kits (Biorbyt; REF: orb564112) were used to detect desmosomal proteins. Sera were run at a dilution of 1/40 as per manufacturers’ instructions. Due to sample availability, not all samples could be run on the protein assays, with the number of samples examined made clear for each result.

#### *Commercial ELISAs to detect anti-*DSG*1 and anti-DSG3 autoantibodies*

Anti-DSG1 (Caltag Medsystems, UK; REF: RG-7880EC-D) and anti-DSG3 (Caltag Medsystems, UK; REF: RG-7885EC-D) autoantibody ELISAs were performed with sera diluted at a dilution of 1/100. Standard protocol as outlined by the product supplier was followed and ran using automated machinery (Dynex DSX, Dynex Technologies Limited, UK).

#### *In-house ELISA to detect anti-* DSG*2 autoantibody*

To detect anti-DSG2 autoantibodies, an in-house ELISA was used. Human DSG2 protein (Biorbyt, UK; REF: orb624074) was diluted to a concentration of 1 µg/ml in Dulbecco’s PBS and 50 μl diluted antigen added to each well of a 96-well plate. The coated 96-well plate was incubated overnight at 4°C. Residual antigen was removed, and the plate was blocked with 200 μl 2% bovine serum albumin (BSA) diluted in PBS for 1 hour, before washing twice with 0.05% PBS-Tween (PBS-T). Each serum sample was diluted 1/100 with 2% BSA in PBS, and 100 μl of diluted sample was added to each well for 1 hour at room temperature with gentle shaking. The plate was then washed four times with 0.05% PBS-T. Anti-human-IgG-HRP conjugate (University of Birmingham, UK) was diluted to 1/12,000 with 2% BSA in PBS and 100 μl diluted HRP-conjugate was added to each well before the plate was incubated for 30 minutes at room temperature with gentle shaking. The plate was then washed four times with 0.05% PBS-T before the addition of 100 μl of 3,3ʹ,5,5ʹ-tetramethylbenzidine (TMB; Bio-Rad Laboratories Inc., USA; REF: BUF056B) which was incubated for 20 minutes at room temperature with gentle shaking. Finally, 100 μl of 0.2 M sulphuric acid was added to each well, and the plate was read using a microplate reader with a 450 nm filter, and the results expressed as the optical density (OD). The protocol was run using automated machinery (Dynex DSX, Dynex Technologies Limited, UK).

### Immunohistochemistry to examine DSG2 staining patterns and the presence of IgG antibody on post-mortem cardiac tissue

Immunohistochemistry was undertaken on cardiac tissue from three post-mortem samples from individuals who died from non-COVID-19 causes and eight individuals who died from COVID-19. Lung tissue from the COVID-19 patients was also examined to rule out non-specific staining. Samples were stained using a mouse anti-DSG2 antibody (AH12.2: sc-80663, Santa Cruz Biotechnology, Texas, USA) by standard IHC techniques. Formalin fixed, paraffin embedded samples were deparaffinised and rehydrated and following low-temperature retrieval (ALTER) [14], immunostained on a Dako Autostainer. Anti-DSG2 antibody was applied for 1 hour at 2μg/ml and visualised with Vector Excel mouse kit and ImmPACT NovaRED (2B Scientific). HRP conjugated goat anti-human pan IgG (2040-05, SouthernBiotech) at 2 μg/ml was applied for 1 hour and visualised with Vector ImmPACT DAB (2B Scientific) to detect any local deposition. Representative images are taken from central portions of the block.

### Statistical Analysis

All results are recorded as median with interquartile range (IQR) where *n* represents numbers of individual samples. Comparisons between two groups were made using the Mann Whitney *U* test. Multiple comparisons were made using the Kruskal-Wallis test with Dunnet’s multiple comparisons post-hoc test.

## Results

### Increased serum DSG2 protein levels selectively associate with acute, severe COVID-19

Desmoplakin (DSP) protein was detectable and at higher levels in both acute COVID-19 ITU patients (86% [*n* = 18/21], 4.08 ng/ml [2.45–6.25]) and individuals admitted to the ITU for reasons other than COVID-19 infection (68% [*n* = 13/19], 1.66 ng/ml [0.94–4.31]) compared with healthy controls (42% [*n* = 13/33], 0.95 ng/ml [0.50–1.61], *P* = 0.0004) (Fig. 1A) Junction plakoglobin (JUP) protein levels found no discernible pattern that discriminated COVID-19 infection or acute disease across the cohorts (Fig. 1B) along with DSG1 and DSG3 protein levels (data not shown). DSG2 protein was most likely to be detected, and at the highest concentration, in individuals from the acute COVID-19 ITU group (Fig. 1C), compared to individuals admitted to the ITU for reasons other than COVID-19 infection and healthy controls (48% [*n* = 10/21], 298 pg/ml [208–633] for the COVID-19 ITU group, 16% [*n* = 13/19], 157 pg/ml [57.2–215], *P* = 0.0007 for non-COVID-19 ITU group, and 21% [*n* = 7/33], 222 pg/ml [140–332], *P* = 0.0068 for healthy controls). This suggests a potential relationship between DSG2 and SARS-CoV-2 infection in more severe COVID-19 presentations.

### Indirect immunofluorescent staining was more likely to be associated with anti-DSG2 rather than anti-DSG1 or 3 autoantibodies

**Figure 2.**
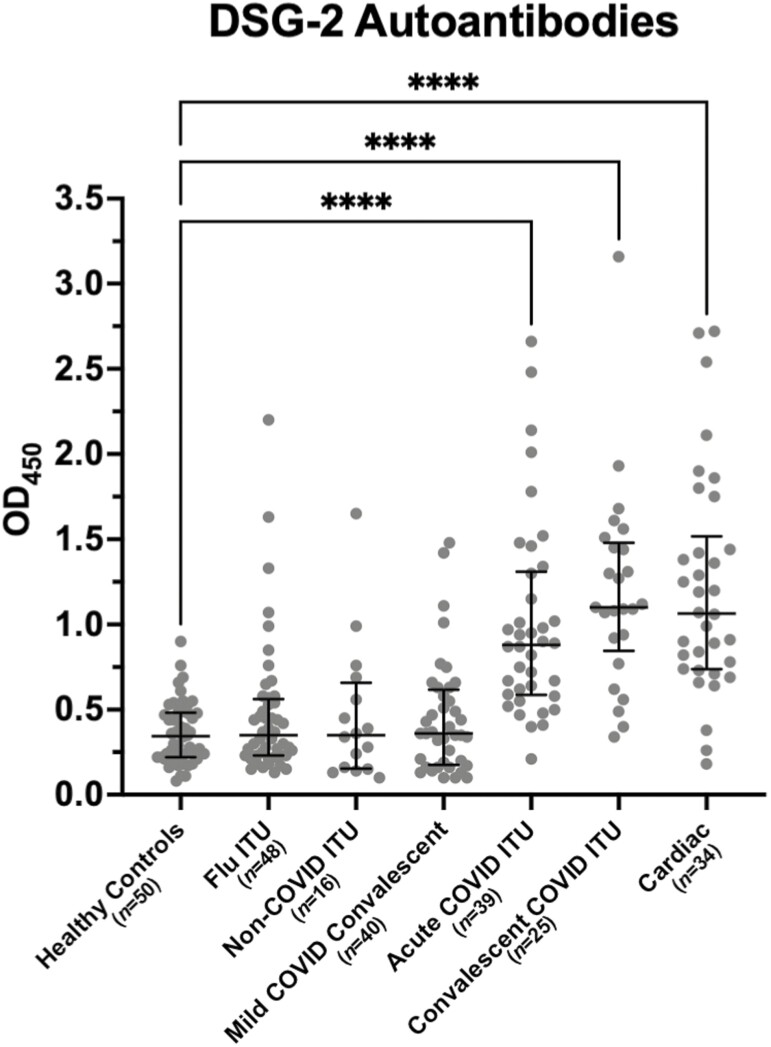
Desmoglein 2 IgG autoantibody concentrations are higher in patients post-severe SARS-CoV-2 infection. Detection of desmoglein 2 autoantibodies in COVID-19 cohorts by ELISA; convalescent COVID Intensive therapy unit (ITU), acute COVID ITU, and mild COVID convalescent, compared with non-COVID control cohorts; non-COVID ITU, Influenza (flu) ITU, and healthy controls (*N* = 252, *P* < 0.0001). The flu ITU, non-COVID ITU, and mild COVID convalescent cohorts were all not statistically significant compared to healthy controls. Results are expressed as median OD (optical density) with interquartile range. Figure in brackets indicates number of sera per group. **** indicates a significant difference between groups at a *P* value of <0.0001.

We found an increased presence of skeletal, cardiac, and epidermal autoantibodies in the acute COVID-19 ITU group, with 23%, 8%, and 46% patients positive, respectively (Table 1, and representative cardiac and epidermal antibody stain shown in Supplementary Fig. S1). These high levels were also observed in convalescent COVID-19 ITU patients where positivity increased to 30% skeletal, 40% cardiac, and 52% epidermal autoantibodies. In comparison, cardiac autoantibodies were the only autoantibodies detected in 6% non-COVID-19 ITU patients with low levels also observed in patients with influenza. The cardiac samples had no evidence of anti-cardiac antibodies to suggest their disease or DSG2 level might be related to ARVC. As intercellular cement skin autoantibodies are associated with anti-DSG1, 2, and 3 autoantibodies, we wanted to determine which DSG autoantibodies the immunofluorescence findings were associated with. We tested the 30 samples from the COVID-19 groups that were positive for skin autoantibodies (intercellular cement staining) by immunofluorescence and found none of these samples were positive for anti-DSG1 or anti-DSG3 autoantibodies. In contrast, we found 80% (*n* = 24/30) had DSG2 autoantibody levels higher than the top interquartile range seen in the healthy control cohort (median OD=0.35 [0.22–0.48], Fig. 2)

### DSG2 autoantibodies are detected selectively in patients with severe COVID-19

To investigate whether the observed increase in DSG2 protein was associated with DSG2 autoantibody production, we developed an in-house ELISA to detect DSG2 IgG autoantibodies. Levels of DSG2 IgG autoantibodies were significantly elevated in sera from both acute (median OD = 0.90[0.60–1.26], *n* = 39, *P*<0.0001) and convalescent (median OD = 1.10[0.85–1.48], *n* = 25, *P* < 0.0001) COVID-19 cohorts following severe disease when compared with healthy controls (median OD = 0.35 [0.22–0.48], *n*=50) (Fig. 2). Sera from the cohort of convalescent individuals following mild disease did not have elevated DSG2 autoantibody levels (median OD = 0.37 [0.19–0.58], *n* = 40, *P* > 0.9999), nor did sera from a non-COVID-19 cohort of ITU patients (median OD = 0.35 [0.16–0.71], *n* = 16, *P*>0.9999), nor sera from a cohort of patients in convalescence after influenza infection (median OD = 0.35 [0.23–0.56], *n* = 48, *P* > 0.9999).

### DSG2 autoantibodies are increased in patients with underlying cardiac comorbidities

Due to the increased rate of autoantibodies against cardiac tissue in our COVID-19 cohorts, we assessed levels of DSG2 autoantibodies in sera from patients with underlying cardiovascular conditions. These studies found antibody levels similar to those found in the COVID-19 patients with severe disease (median OD = 1.07 [0.74–1.44], *n* = 34, *P* < 0.0001) (Fig. 2). The type of cardiac comorbidity, nor severity of disease as stratified by ejection fraction, Troponin T, NT-proBNP, IL-6 nor hsCRP, did not predict the presence of DSG2 autoantibodies. We obtained history on previous cardiac comorbidity in each of the groups examined (Table 1), but found no relationship with the raised DSG2 autoantibody levels observed in the severe COVID-19 patients in both the acute and convalescent setting.

### Intercalated disc staining for DSG2 is abnormal in COVID-19 post-mortem cardiac tissues

**Figure 3.**
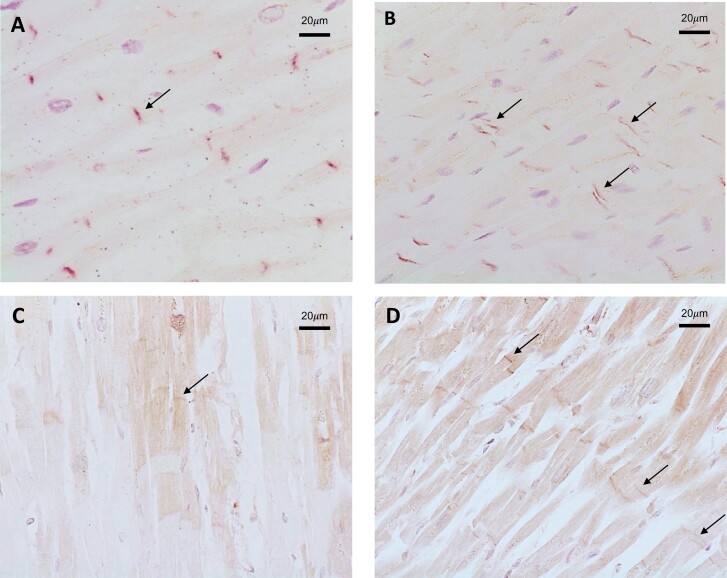
Immunohistochemical staining of post-mortem cardiac tissue. (**A**) Non-COVID-19 post-mortem heart tissue shows uniform single-banded staining for DSG2 corresponding to the site of intercalated discs (these are indicated by black arrows). (**B**) Cardiac muscle from COVID-19 post-mortem cases showed areas of single and notable double banded staining for DSG2 (arrowed). (**C**) Post-mortem heart tissue from a non-COVID-19 patient stained for IgG deposition. (**D**) Post-mortem heart tissue from a COVID-19 patient demonstrating IgG deposition at intercalated discs. Images representative of 3 hearts from non-COVID-19 patients and 8 patients who died from COVID-19.

All hearts from individuals who did (*n* = 8) or did not (*n* = 3) die from COVID-19 causes (*n* = 3) showed variable weak to strong staining of DSG2 corresponding to the site of the intercalated discs (Fig. 3). Areas were also observed with no staining due to post-mortem degradation of the tissues. In the non-COVID-19 post-mortem heart tissues DSG2 staining consistently presented as a single, distinct band (Fig. 3A). In the post-mortem heart tissues from COVID-19 positive patients (*n* = 8) also showed a similar staining pattern, but in addition, there were distinct areas of double-banded staining observed, which are indicative of the separation of the intercalated disc [15] (Fig. 3B). Importantly, this distinct ‘double’ banding pattern was not seen in non-COVID-19 cardiac muscle, even when there was discontinuation of the muscle as a result of post-mortem degradation. No DSG2 staining was observed in the post-mortem lung tissue (*n* = 8) examined from COVID-19 patients. Finally, we determined if IgG was detectable on cardiac tissue and intercalated discs by staining with an anti-human IgG antibody. This showed that IgG was observed at the site of the intercalated discs of hearts from patients who died from COVID-19.

## Discussion

In this study, we have found raised DSG2 protein and a higher frequency of DSG2 autoantibody production in patients with or following severe COVID-19. Increased anti-DSG2 autoantibodies are not induced to all infections as evidenced by the relative absence of anti-DSG2 responses in other groups including a cohort of influenza patients. Furthermore, the detection of perturbed DSG2 staining in post-mortem human heart tissue from COVID-19 patients reveals structural changes of the cardiomyocyte intercalated discs which are rich in DSG2.

There is an extensive literature on the association between numerous viral infections and autoimmunity. This can be due to a number of mechanisms including molecular mimicry, epitope spreading, and bystander activation. COVID-19 is no exception with clinical syndromes such as autoimmune cytopenia, Guillain-Barre syndrome, autoimmune encephalitis, lupus, and acquired haemophilia being reported [16]. COVID-19 infection results in the post-translational modification of a vast number of proteins with high-intrinsic propensity to become autoantigens, offering an explanation for the diverse autoimmune complications observed [17].

Although low-titre, transient autoantibodies occur with many acute viral infections [18] their clinical significance and pathogenic potential are uncertain. We have previously reported an increase in autoantibodies post-COVID-19 [1] with a specific pattern of autoantibodies against cardiac, skin, and muscle and shown that these were still present at 6 months post-infection. Similarly, in this study, we find an increased rate of DSG2 autoantibodies, and that these persist to 6 months following an episode of severe COVID-19. These are unlikely to be present transiently due to a single episode of infection as the half-life for IgG is approximately 3 weeks. The potential for chronicity of these antibodies is further supported by the serological response being similar between the chronic COVID-19 group, the acute group and the cardiac group. Nevertheless, further studies, particularly longitudinal studies, are necessary to provide more conclusive insights as alternative interpretations of these data cannot be ruled out at this stage. The finding of autoantibodies at 6 months raises the possibility that there may be an ongoing clinical consequence; however, future study is required of longitudinal samples with clinical follow up data to establish causation.

DSG2 autoantibodies have the potential to be pathogenic and have been associated with ARVC and familial dilated cardiomyopathy [7]. Furthermore, DSG2 autoantibodies have been shown to disrupt DSG2 protein function with the consequence of interfering with cell-to-cell adhesion [7, 19]. Consequent intercellular space widening at the level of the intercalated disc (desmosomes/adherens junction) and reduction in action potential velocity results in the increased arrhythmia susceptibility observed [20].

Cardiac injury following COVID-19 infection is now well-described in the literature, although the mechanisms behind this remain uncertain [11]. Clinical studies have reported post-COVID-19 complications such as thromboembolism, ischaemia, arrhythmias, conduction defections, and myocarditis [21–23] and post-mortem studies have found increased neutrophil extracellular trap (NET) formation and mononuclear cell infiltration [13]. Given that cardiac involvement is an important and long-term consequence of COVID-19, and potential also within the spectrum of syndromes observed in ‘long-COVID’, we examined post-mortem heart tissue for DSG2 protein. We found that DSG2 is localized to the intercalated discs, confirming previous studies, and importantly that these discs were only found to be widened in COVID-19 tissue samples as is seen in ARVC [8, 20]. Previous in vitro studies for ARVC have found that inhibition of DSG2 binding, or mutation of DSG2 protein, disrupts intercalated discs and subsequently the cell to cell contact necessary for cardiomyocyte adhesion. It is increasingly recognized that DSG2 is a multifunctional protein and may have a role in carcinogenesis [24], angiogenesis and re-localization of actin [25], early haematopoietic development in the bone marrow, in particular myeloid progenitors [25], intestinal epithelial apoptosis, and haemostasis [26]. Finally, DSG2 has been identified as the primary high-affinity receptor used by a number of adenovirus (Ad) serotypes which cause severe respiratory disease in humans [27, 28].

The presence of these autoantibodies may be indirect markers of historic tissue damage or alternatively they may play a pathogenic role by disrupting intercalated discs and, therefore, normal cardiac function. The visualization of IgG deposition on intercalated discs further raises the possibility of antibody interference. Future studies will have to examine how DSG2 autoantibodies may interfere with DSG2 protein function and the clinical consequences of this and replicated in larger studies with clinically associated data, this may offer a potential biomarker for the multiple and diverse long-term sequelae following COVID-19 infection.

In conclusion, the findings of DSG2 autoantibodies and intercalated disc widening offer a potential autoimmune mechanism for some of the complications found post-COVID-19 infection. Further studies in well characterized clinical cohorts, with comorbid complications, are required to prove whether the presence of DSG2 antibodies serves as a marker of post-acute COVID-19 syndrome or long COVID. Detailed mechanistic studies are required to reveal the link between autoimmunity to DSG2 and the long-term sequelae of SARS-CoV-2 infection.

## Supplementary Material

uxad046_suppl_Supplementary_Figure_1

## Data Availability

The data underlying this article will be shared on reasonable request to the corresponding author. Pre-print available on Medrxiv at https://doi.org/10.1101/2022.07.26.22278002

## Abbreviations

ARVC: arrhythmogenic right ventricular cardiomyopathy
BSA: bovine serum albumin
COCO Study: Covid-19 convalescent immunity study
DSG: desmoglein
DSG2: desmoglein 2
DSP: desmoplakin
ELISA: enzyme-linked immunosorbant assay
HRP: horseradish peroxidise
IHC: immunohistochemistry
IQR: interquartile range
ITU: intensive therapy unit
JUP: junction plakoglobin
LoST-SoCC Study: Long land short-term effects of Standard-of-Care treatments for Cancer study
NET: neutrophil extracellular trap
OD: optical density
PBS: phosphate buffered saline
PBS-T: PBS-Tween
PV: *Pemphigus vulgaris*
TMB: 3,3ʹ,5,5ʹ-tetramethylbenzidine.
