# Supplementary figures and images for: SARS-CoV-2 infection is associated with anti-desmoglein 2 autoantibody detection
Source: Clin Exp Immunol. 2023 Apr 24;213(2):243–51. doi: 10.1093/cei/uxad046 (PMC10651225; doi:10.1093/cei/uxad046)

## Slide 1
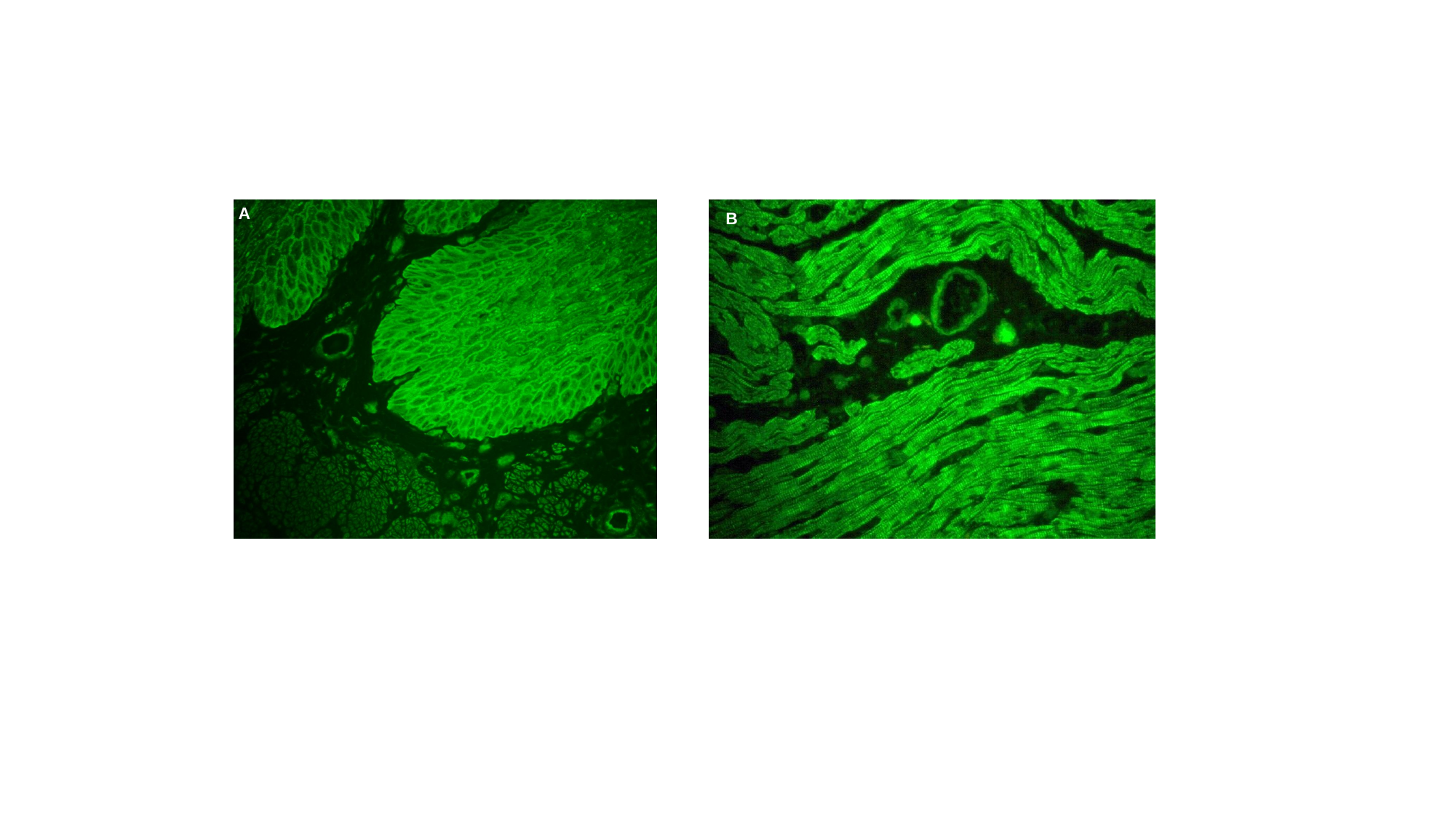

A
B

Supplement: uxad046_suppl_Supplementary_Figure_1 [file uxad046_suppl_supplementary_figure_1.pptx]
